# Money Protects White but Not African American Men against Discrimination: Comparison of African American and White Men in the Same Geographic Areas

**DOI:** 10.3390/ijerph18052706

**Published:** 2021-03-08

**Authors:** Shervin Assari, Susan D. Cochran, Vickie M. Mays

**Affiliations:** 1 Department of Family Medicine, Charles Drew University, Los Angeles, CA 90059, USA; shervinassari@cdrewu.edu; 2 UCLA BRITE Center for Science, Research and Policy, University of California, Los Angeles (UCLA), Los Angeles, CA 90095-1563, USA; cochran@ucla.edu; 3 Department of Epidemiology, Fielding School of Public Health, University of California, Los Angeles (UCLA), Los Angeles, CA 90095-1772, USA; 4 Department of Statistics, University of California, Los Angeles (UCLA), Los Angeles, CA 90095-1554, USA; 5 Department of Health Policy and Management, Fielding School of Public Health, University of California, Los Angeles (UCLA), Los Angeles, CA 90095-1772, USA; 6 Department of Psychology, University of California, Los Angeles (UCLA), Los Angeles, CA 90095-1563, USA

**Keywords:** discrimination, perceived discrimination, socioeconomic status, income, education, racism

## Abstract

To compare African American (AA) and non-Hispanic White men living in same residential areas for the associations between educational attainment and household income with perceived discrimination (PD). The National Survey of American Life (NSAL), a nationally representative study, included 1643 men who were either African American (*n* = 1271) or non-Hispanic White (*n* = 372). We compared the associations between the two race groups using linear regression. In the total sample, high household income was significantly associated with lower levels of PD. There were interactions between race and household income, suggesting that the association between household income and PD significantly differs for African American and non-Hispanic White men. For non-Hispanic White men, household income was inversely associated with PD. For African American men, however, household income was not related to PD. While higher income offers greater protection for non-Hispanic White men against PD, African American men perceive higher levels of discrimination compared to White males, regardless of income levels. Understanding the role this similar but unequal experience plays in the physical and mental health of African American men is worth exploring. Additionally, developing an enhanced understanding of the drivers for high-income African American men’s cognitive appraisal of discrimination may be useful in anticipating and addressing the health impacts of that discrimination. Equally important to discerning how social determinants work in high-income African American men’s physical and mental health may be investigating the impact of the mental health and wellbeing of deferment based on perceived discrimination of dreams and aspirations associated with achieving high levels of education and income attainment of Black men.

## 1. Introduction

While socioeconomic status (SES) indicators such as educational attainment and household income protect most populations from poor health [1], African Americans (AA) experience smaller returns from their SES relative to Whites [2], a phenomenon called Minorities’ Diminishing Returns (MDRs) [3,4]. For African American individuals, restricted protective effects of education attainment and income have been shown for a wide range of physical and mental health outcomes [2]. These patterns are robust as they are observed for children, youth, adults, and older adults, and replicated regardless of cohorts and settings [2]. One manifestation of MDRs is that high education and income may operate as risk factors for depression for African American men and boys [5].

As noted in the “Black –White wealth gap” arguments by Oliver and Shapiro [6] as well as Hamilton and Darity [7], a considerable racial wealth gap persists between African American and White individuals with similar education and income profiles. This has sometimes been alluded to as the Black tax, which refers to the hidden costs of being African American in the US [8]. While much work has focused on the contribution of institutional and structural racism in the economic and labor market in reducing the benefits of educational and income attainment for African American individuals, less work has focused on the role of perceived discrimination (PD) in this regard. We know even less about the experiences of African American men who despite PD manage to acquire high levels of education and income [9].

At least for three reasons, PD might be one of the plausible mechanisms that can explain diminished returns of SES for African Americans who manage to obtain high levels of education and income [2,9]. First, PD is one of the major contributors of racial/ethnic health disparities in the United States. As shown by multiple review papers [10,11], PD increases risk of multiple physical and mental health outcomes. Second, some evidence suggests that high-SES African American people report more, not less PD [9], which is in part due to an increased contact with Whites [12]. High-SES African American individuals who live in a predominantly White context may also have less access to the African American community, which could provide social support. Third, research suggests that the social patterning of PD is different for African American and White individuals [12,13,14]. While for White Americans, PD is less common among high-SES individuals [15], high-SES African American individuals report more, but not less, PD, compared to their low-SES counterparts [9,13].

Although PD can be considered a plausible explanation for MDRs, there are very few studies that have specifically compared non-Hispanic White and African American individuals for differences in the associations between SES and PD. In a recent study, Colen and colleagues [13] used data from the National Longitudinal Survey of Youth (NLSY). For Whites, income gain over time was associated with less exposure to chronic discrimination. Upwardly mobile African American people, however, reported more PD compared to their socioeconomically stable counterparts. In this study, differential exposure to PD explained a substantial proportion of the gap between African American and White individuals in self-rated health [13]. Most other studies suggesting that high-SES African American people experience high levels of discrimination [14] have been limited to African American individuals only; thus, they do not provide information on differential effects of educational attainment or income on PD between non-Hispanic White and African American people, particularly those living in the same areas. The positive association between SES and PD among African American individuals may be more pronounced for males, probably because African American men are the main target of discrimination by non-Hispanic White men [16].

### Aims

To better understand the role of PD in MDRs [2,3,4], this study used a national sample to compare African American and non-Hispanic White men for the associations of education attainment and household income with PD. We hypothesized inverse associations between educational attainment and income with PD for non-Hispanic White men but not for African American men.

## 2. Materials and Methods

### 2.1. Design

Data from the National Survey of American Life (NSAL-Adults), a nationally representative mental health survey of non-Hispanic White and African American adults [17], were used. Although NSAL methodology is well explained elsewhere [17], we briefly describe the study here.

### 2.2. Participants and Sampling

The NSAL used household probability sampling methods to draw a nationally representative sample of African American and non-Hispanic White adults who reside in the same areas [17]. The multistage sampling design produced a core national sample of White and African American adults, almost identical to the earlier National Survey of Black Americans (NSBA). In NSAL, non-Hispanic White and African American participants were selected from the same context and geographic areas, so non-Hispanic Whites are representative to the geographic areas of the US in which there are at least 10% African American residing. In other words, the non-Hispanic White sample in the NSAL is not representative of all non-Hispanic Whites in the U.S. as they are drawn from proximity to African American people compared to all non-Hispanic Whites in the U.S. [18]. Participants were adults (age ≥ 18 years) who lived in the coterminous U.S. (48 states). All were non-institutionalized individuals who could complete a structured interview administered in English. These excluded individuals residing in long-term medical care settings, nursing homes, prisons, or jails [18]. We restrict our analytical sample to the 1643 male participants who were either non-Hispanic White (*n* = 372) or African American (*n* = 1271).

### 2.3. Data Collection

The NSAL administered a structured interview in English. About 82% of total interviews were face-to-face, while the remaining 14% were conducted over the telephone. Computer-assisted personal interview (CAPI) methods were used. These methods enhance data quality in long and complex surveys such as the NSAL due to its complex skip patterns. Interviews averaged 140 min in length. The response rate was 71% for African American and 70% for non-Hispanic White respondents.

### 2.4. Measures

For the current study, we used the following variables from the NSAL:

#### 2.4.1. Race/Ethnicity

In the NSAL, race/ethnicity was self-identified. For the current study, all participants identified themselves as either African American or non-Hispanic White. African American participants were restricted to individuals without any ancestral ties to the Caribbean countries.

#### 2.4.2. Educational Attainment

Educational attainment was measured using self-report. Levels of educational attainment were coded as: (1) 11 years or less (less than high school diploma), (2) 12 years (high school graduate), (3) 13–15 years (some college but did not complete college), and (4) 16+ years (college graduate). We treated educational attainment as three dummy variables: (1) 12 years (high school graduate), (2) 13–15 years (some college but did not complete college), and (3) 16+ years (college graduate). As such, our omitted category was less than high-school diploma (11 years or less). For sensitivity analysis, we used educational attainment as a continuous measure (years of schooling).

#### 2.4.3. Household Income

Household income was assessed using self-report. Annual household income was collected as a continuous measure, which had a normal distribution. We used quartiles for the total sample, regardless of race/ethnicity, rather than race/ethnic-specific income thresholds, so similar to education attainment, income levels were comparable across race/ethnic groups. The thresholds were as bellow: 15,000 (1.4–1.5) for the 1st quantile, 28,000 (27,500–29,000) for the 2nd quantile (50th percentile), and 47,000 (45,000–48,000) for the 3rd quantile (75th percentile). We included three dummy variables for data analysis with missing category being the lowest income level. For sensitivity analysis, we used income as a continuous measure.

#### 2.4.4. Perceived Discrimination (PD)

PD was measured using David Williams’ Everyday Discrimination Scale (EDS) [18]. This scale uses ten items to assess routine, chronic, daily, and less overt discriminatory experiences over the past year [19]. Sample items include “In your day-to-day, life how often have any of the following things happened to you?” Sample items include: “being followed around in stores”, “people acting as if they think you are dishonest”, “receiving poorer service than other people at restaurants”, and “being called names or insulted”. Responses are given on a Likert scale ranging from 1 (“never”) to 6 (“almost every day”). For the 10 items, we calculated a summary score ranging from 0 to 50; a higher score reflects more frequent experiences with discriminatory events over the past year (Cronbach’s α in this study = 0.86).

### 2.5. Statistical Analysis

We used Stata 16.0 to analyze the data taking into account the complex sampling design and study weights. As a result, analytic inferences and rates are generalizable to the US population of similar adult men. To prepare for linear regression analyses, we first evaluated possible multicollinearity between educational attainment and household income particularly among the Non-Hispanic White sample where education and income are likely to have a stronger correlation. We also tested the assumption of linearity of the distribution of residuals (errors) before we fit our linear regression models. In all the models that we estimated, educational attainment (three dummy variables) and household income (three dummy variables) were the main independent variables, perceived (daily) discrimination was the dependent variable, and age, marital status, employment status, and household size were the covariates. We decided to control for these variables because African American men are more likely to be unemployed, be unmarried, and live with a higher number of individuals in the household.

We first report results of descriptive analyses comparing males by race. For these comparisons, we used unadjusted regression analysis, which is more robust than t test to unequal variance (due to imbalanced sample size across racial groups). Next, we estimated linear regressions regressing PD on our two major SES predictors, educational attainment, and household income, treated as dummy variables, while controlling for confounding due to age, marital status, employment status, and household size. We did this in 4 steps. First, we used the pooled sample to estimate a regression model predicting PD reports using main effects of race, educational attainment, household income, and covariates but no interaction terms (Model 1). Next, we added interactions to our regression model: educational attainment × race and household income × race/ethnicity (Model 2). We then estimated similar stratified models for African American (Model 3) and Non-Hispanic White (Model 4) men separately. For sensitivity analyses, we treated educational attainment and household income as interval variables. As the results lead to identical inferences, we did not report the results of replication here; however, they are available from the authors. We reported regression coefficients (b), their standard errors (SEs), associated 95% confidence intervals (CIs), and p-values. A p-value of less than 0.05 was considered significant.

## 3. Results

### 3.1. Descriptive Statistics

Of the 1643 participating men who entered this analysis, 1271 were African American and 372 were Non-Hispanic White. As shown in Table 1, African American men were younger, had lower educational attainment, were less likely to be married, and were more likely to be unemployed and reported lower household income than White men were. African American men also reported higher levels of PD than Non-Hispanic White men.

### 3.2. Pooled Sample

In Table 2, we summarize the results of two linear regressions with PD as the outcome. Results from Model 1 indicated that being African American as opposed to being Non-Hispanic White, being younger, and reporting lower levels of household income were associated with higher level of PD. Educational attainment, however, was not associated with PD.

When interaction terms were added to the model (Model 2), the main effect of race/ethnicity attenuated with the variance of the effect being reflected in the interaction terms between race/ethnicity and household income. Comparisons of estimate means that higher income has a larger protective (inverse) effect on PD for Non-Hispanic White men than African American men.

### 3.3. Stratified Models

In Table 3, we present the results of the race-stratified regression models. Among African American men (Model 3), high household income was uncorrelated with PD (*p* = 0.71). For White men (Model 4), however, higher household income was associated with less PD (b = −0.91 *p* = 0.009). For none of the groups was educational attainment associated with PD (*p* > 0.05 for both races).

### 3.4. Robustness Check

**Figure A1:**
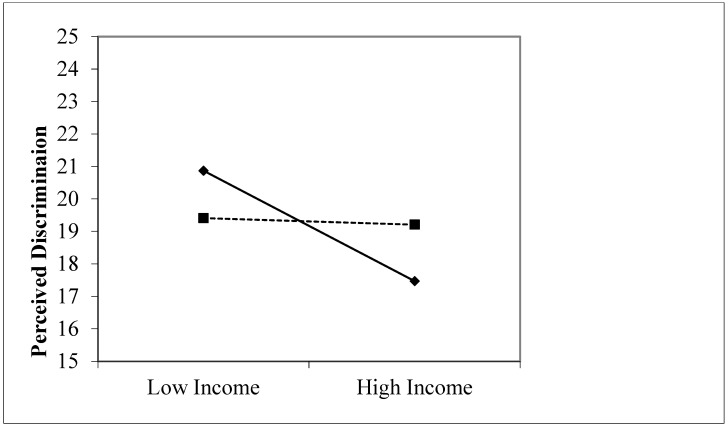
Interaction between race and income on perceived discrimination in White (solid line) and African American (dotted line) men. Source: National Survey of American Life.

While our main models used parental education and household income as three dummy variables, we also ran replication models with educational attainment and household income as continuous/interval measures. Similar to our main analysis, education attainment did not have a main effect or interaction with race/ethnicity, while household income had a protective effect and also showed interaction with race/ethnicity, suggesting weaker protective effect of household income for African American than Non-Hispanic White men (Figure A1, Table A1 and Table A2).

## 4. Discussion

Two major results were found. First, high household income but not high educational attainment was protective against PD in the overall sample of American men. Second, a protective effect of household income against PD was only detectable for Non-Hispanic White but not African American men. That is, although high-income Non-Hispanic White men are protected against PD, African American men report high levels of PD at all income levels.

At least three recent studies have documented related findings. In the first study, Colen and colleagues [13] showed that for Whites, income gain over time was associated with less exposure to PD. Upwardly mobile African American people, however, reported more PD compared to their socioeconomically stable counterparts [13]. In the second study, while income improved self-rated mental health for Whites, African American individuals reported poor self-rated mental health across all income levels [20]. A third study showed that upward and downward educational mobility were associated with an increase in stressful life events for Non-Hispanic Whites; however, African American individuals reported high levels of stressful life events, regardless of their social mobility status [21]. Thus, for African American individuals, various types of stress are not reduced as a result of occupying a higher status in society. Instead, race/ethnicity appears to serve as a steady magnet for discrimination regardless of social position. Thus, the stress of race-based discrimination should be regarded as one of several plausible explanations for MDRs [9]. Studies have demonstrated that this type of stress is detrimental to the mental health of African American men, particularly those with high hegemonic masculine beliefs [22].

Constantly high levels of PD across all income levels for African American men suggest that, in contrast to Non-Hispanic White men for whom poverty and lack of materialistic resources are primary causes of discrimination [15], African American men’s PD is mainly a function of their race/ethnicity (i.e., the social position due to being African American) and not the accumulation of materialistic resources [9,15]. Gendered and racist stereotypes simultaneously affect various aspects of African American men’s lives [23]. Racism and discrimination are embedded in the fabric of the US society, which affects African American men’s daily lives across settings and institutions. Hutchinson has argued that African American men suffer a unique oppression because of their sex/gender [24]. Gendered racism, anti-Black misandry, and outgroup male discrimination could be underlying factors that contribute to the existing disparities in PD observed in this study [24]. Chetty recently documented that upward social mobility is least likely for African American men [25]. In another recent study, education attainment protected African American women but not men against psychological distress and depression [26].

The current study is not the first to document diminished returns of income for African American men. In their book *Black Wealth/White Wealth* [6,8] and the discussion of the Black tax [8], Shapiro and Oliver argued that African American individuals often gain fewer tangible outcomes than Non-Hispanic White Americans even when their income is the same. Darity and Hamilton have also documented extensive wealth gap between Non-Hispanic Whites and African American individuals [7]. The same is true for the effect of higher education in African American people not resulting in similar occupational achievement or salaries compared to Non-Hispanic Whites [6,8]. Some research has suggested that educational systems may foster inequalities rather than eliminate them; grade attainment remains an elusive equalizer in the US for African American men, despite its continued use in assessing differences in SES [27]. Some of these observed differences may be due to the societal privilege and power associated with Whiteness [28].

Williams [29] and Farmer and Ferraro [3] have shown that racial gaps are largest at the highest rather than lowest social positions, which helps us to understand why high income is less protective for African American men. Navarro argued that it is “race and class” rather than “race or class” that shapes racial inequalities in the US [30]. Thus, it is not just lack of materialistic resources or poverty but also the added burden of race-based discrimination that results in disparities. It is not either class or race, but their intersections that shape societal privilege and power [31,32]. It is the intersection of race, gender, and class, which gives income its purchasing power [31,32]. As Wilson, Thorpe, and Laviest [4] as well as Oliver and Shapiro [6,8] have argued, while all money may be green, major racial differences exist in its purchasing power.

As Williams has proposed, discrimination may be why high levels of education generates less income and health for African American than Non-Hispanic White individuals [3,33]. More research is, however, needed on the differential effects of discrimination based on the societal position, which follows the intersections of intersections of race, gender, and class (e.g., educational attainment and income).

### Limitations

Our findings should be interpreted in the context of some study limitations. First, the cross-sectional design of our study does not imply causation but association. While SES may affect PD, it is also possible that factors that interfere with upward social mobility also affect perception or experience of discrimination. Second, this study did not distinguish type and source of discrimination. Discrimination due to race, SES, gender, and age may have differential effects. Third, the current study could not control all potential confounders. Our study was not able to speak to the experiences of very high-income level individuals/households because they were not captured in the National Survey of American Life. Fourth, this study used household rather than personal income. While the unit of analysis in this study was household rather than the individual, household income may better reflect all the resources that a person has to bring to bear in their social position.

## 5. Conclusions

Focusing the research agenda to answer the many unanswered research questions raised in our study would enhance efforts to better identify and address the unique ways that African American men face higher levels of negative mental and physical health outcomes in the United States. Our results showed that social patterning of PD based on household income differs for African American men from that of Non-Hispanic White men. That is, race/ethnicity and income have nonlinear and intersectional effects on the distribution of PD, with higher-income African American men not having equal uplifting of their disadvantage in ways Non-Hispanic White men benefit from income and education Our findings may potentially explain why African American men do not gain much physical and mental health protection against negative health outcomes in the face of increasing attainment of income and education. In the United States, where the ideology of individualism is prevalent, it is believed that hard work in the face of opportunity can facilitate an individual reaching the American dream. It is believed that through the attainment of higher levels of income and educational attainment, greater relief will be found against being treated differently, badly, or unfairly [34]. This belief carries with it the idea that pathways to achievement can be accomplished regardless of race, class, gender, age, or national origin [34]. Results of our study show that this is clearly not the case for many African American men. It is therefore important to acknowledge that the path to reducing health disparities for African American men cannot be only through individual economic or behavioral changes alone but must address the subtle and not so subtle ways in which structural barriers limit and bound the benefits of African American men’s economic achievements. African American men’s dreams deferred is an experience that should be examined relative to their poor physical and mental health outcomes [35].

## Figures and Tables

**Table 1:** Descriptive characteristics in the pooled sample by race among non-Hispanic African American and Non-Hispanic White men in the National Survey of American Life.

Sociodemographics & PD	Total Sample (*n* = 1643)		African American Men (*n* = 1271)		Non-Hispanic White Men (*n* = 372)	
	Proportion (SE)	95% CI	Proportion (SE)	95% CI	Proportion (SE)	95% CI
NHW (Non-Hispanic White)	53.23 (3.45)	46.28–60.05				
AA (African American)	46.77 (3.45)	39.95–53.72				
Household Income *						
1st quantile	15.60 (1.54)	12.74–18.96	20.19 (1.70)	16.95–23.87	11.57 (2.47)	7.25–17.96
2nd quantile	18.83 (2.16)	14.87–23.55	18.91 (1.19)	16.61–21.46	18.75 (3.92)	11.77–28.53
3rd quantile	27.42 (2.20)	23.22–32.05	30.01 (1.51)	27.04–33.15	25.14 (3.83)	17.88–34.13
4th quantile	38.16 (3.60)	31.23–45.60	30.89 (2.16)	26.69–35.44	44.54 (6.41)	31.60–58.27
Educational Attainment *						
0–11 Years	19.27 (1.77)	15.96–23.07	23.11 (1.58)	20.06–26.48	15.88 (3.02)	10.44–23.43
12 Years	36.58 (3.09)	30.62–42.98	39.57 (1.78)	36.02–43.25	33.94 (5.60)	23.18–46.67
13–15 Years	22.72 (1.45)	19.93–25.76	22.95 (1.63)	19.82–26.43	22.50 (2.30)	17.98–27.79
16+ Years	21.44 (3.71)	14.91–29.83	14.36 (1.52)	11.54–17.72	27.67 (6.67)	15.82–43.76
Employment Status *						
Employed	94.27 (0.87)	92.26–95.78	91.24 (1.01)	88.96–93.08	96.93 (1.16)	93.23–98.64
Unemployed	5.73 (0.87)	4.22–7.74	8.76 (1.01)	6.92–11.04	3.07 (1.16)	1.36–6.77
Marital Status *						
Not Married	44.85 (2.55)	39.80–50.00	50.62 (1.65)	47.27–53.96	39.77 (4.31)	31.03–49.23
Married	55.15 (2.55)	50.00–60.20	49.38 (1.65)	46.04–52.73	60.23 (4.31)	50.77–68.97
	Mean (SE)	95% CI	Mean (SE)	95% CI	Mean (SE)	95% CI
Age (Years) *	44.15 (0.76)	42.63–45.68	41.76 (0.65)	40.44–43.09	46.22 (1.31)	43.44–49.01
Household Size *	2.53 (0.09)	2.36–2.70	2.73 (0.06)	2.61–2.86	2.36 (0.15)	2.03–2.69
Perceived Discrimination *	11.21 (0.33)	10.54–11.87	13.76 (0.48)	12.79–14.73	9.00 (0.32)	8.32–9.68

Notes: Source: National Survey of American Life (NSAL 2001–2003), PD: perceived discrimination; CI: Confidence Interval; SE: Standard Error. * *p* < 0.05.

**Table 2:** Summary of linear regression on the effects of educational attainment and household income on perceived discrimination in the pooled sample of African American and Non-Hispanic White men in the National Survey of American Life.

Sociodemographics	Total Sample (*n* = 1643)					
	Model 1 Main Effects			Model 2 M1 + Interactions		
	b (SE)	95% CI	*p*	b (SE)	95% CI	*p*
All						
Race (African Americans)	3.83 (0.53)	2.76–4.89	<0.001 ***	−1.40 (2.60)	−6.64–3.83	0.592
Age	−0.14 (0.01)	−0.16–0.11	<0.001 ***	−0.14 (0.01)	−0.16–0.11	<0.001 ***
Household Size	−0.10 (0.23)	−0.56–0.37	0.684	−0.03 (0.22)	−0.47–0.40	0.875
Unemployed	2.45 (1.51)	−0.59–5.49	0.112	2.52 (1.45)	−0.40–5.44	0.089
Married	−0.56 (0.46)	−1.48–0.37	0.232	−0.55 (0.47)	−1.50–0.39	0.245
Educational attainment						
0–11						
12 years	−0.38 (0.81)	−2.00–1.24	0.639	−0.65 (1.14)	−2.93–1.63	0.571
13–15 years	1.14 (1.04)	−0.94–3.23	0.275	0.72 (1.63)	−2.57–4.00	0.662
16 + years	−0.39 (1.14)	−2.69–1.91	0.732	−0.59 (1.55)	−3.70–2.53	0.705
Household income						
1st quantile						
2nd quantile	−2.58 (1.51)	−5.63–0.46	0.095	−6.02 (2.67)	−11.40–0.65	0.029
3rd quantile	−3.30 (1.24)	−5.79–0.80	0.011 *	−6.31 (2.14)	−10.60–2.02	0.005 **
4th quantile	−2.20 (1.04)	−4.30–0.11	0.040	−5.38 (1.80)	−9.00–1.75	0.004 **
Race × Education (12 years)				0.69 (1.51)	−2.34–3.72	0.648
Race × Education (13–15 years)				1.00 (1.90)	−2.83–4.82	0.604
Race × Education (16+ years)				0.79 (1.88)	−2.99–4.58	0.675
Race × Income (2nd quantile)				6.02 (2.85)	0.29–11.75	0.040
Race × Income (3rd quantile)				4.95 (2.44)	0.05–9.85	0.048
Race × Income (4th quantile)				5.40 (2.07)	1.24–9.56	0.012 *
Intercept	18.01 (1.89)	14.20–21.81	<0.001 ***	20.95 (2.35)	16.22–25.67	<0.001 ***

Notes: Source: National Survey of American Life (NSAL 2001–2003), Outcome: Discrimination (Everyday), Independent variables treated as dummy variables. CI: Confidence Interval; SE: Standard Error. * *p* < 0.05, ** *p* < 0.01, *** *p* < 0.001.

**Table 3:** Summary of linear regression on the effects of education attainment and household income on perceived discrimination by race among men in the National Survey of American Life (NSAL).

Characteristics	Total Sample (*n* = 1643)					
	Model 3 African American Men			Model 4 Non-Hispanic White Men		
	b (SE)	95% CI	*p*	b (SE)	95% CI	*p*
All						
Age	−0.12 (0.02)	−0.15–0.09	<0.001 ***	−0.16 (0.03)	−0.22–0.10	<0.001 ***
Household Size	0.37 (0.20)	−0.04–0.78	0.074	−0.60 (0.46)	−1.57–0.38	0.211
Unemployed	1.51 (1.35)	−1.24–4.26	0.272	4.85 (3.45)	−2.49–12.19	0.180
Married	−1.15 (0.70)	−2.58–0.28	0.113	0.25 (0.70)	−1.24–1.74	0.725
Educational attainment						
0–11						
12 years	0.15 (1.00)	−1.89–2.19	0.884	−0.47 (1.11)	−2.83–1.89	0.679
13–15 years	1.87 (1.02)	−0.20–3.94	0.075	0.76 (1.65)	−2.76–4.28	0.652
16+ years	0.39 (1.11)	−1.86–2.64	0.729	−0.71 (1.62)	−4.16–2.73	0.665
Household income						
1st quantile						
2nd quantile	0.04 (1.02)	−2.03–2.12	0.966	−5.71 (2.75)	−11.58–0.15	0.055
3rd quantile	−1.40 (1.23)	−3.91–1.10	0.263	−6.12 (2.19)	−10.79–1.44	0.014 *
4th quantile	−0.16 (1.27)	−2.73–2.42	0.903	−5.14 (1.93)	−9.25–1.03	0.018 *
Intercept	18.01 (1.55)	14.85–21.17	<0.001 ***	22.51 (2.65)	16.87–28.15	<0.001 ***

Notes: Source: National Survey of American Life (NSAL 2001–2003), Outcome: Discrimination (Everyday), Independent variables treated as dummy variables. Confidence Interval (CI); Standard Error (SE). * *p* < 0.05, *** *p* < 0.001.

## Data Availability

This data is available through the University of Michigan ICPSR.

## Appendix A

**Table A1:** Summary of linear regressions on the effects of educational attainment and household income as interval variables on perceived discrimination in African American and Non-Hispanic White men in the National Survey of American Life (sensitivity analysis).

Characteristics	African American Men (*n* = 1271)			White Men (*n* = 372)		
	Model 3			Model 4		
	b (SE)	95% CI	*p*	b (SE)	95% CI	*p*
Age	−0.14 (0.01) ***	−0.17–0.11	<0.001	−0.15 (0.02) ***	−0.19–0.11	<0.001
Educational attainment	0.31 (0.35)	−0.40–1.01	0.381	−0.14 (0.58)	−1.38–1.10	0.817
Household income	−0.10 (0.27)	−0.66–0.45	0.707	−0.91 (0.31) **	−1.56–0.26	0.009
Intercept	19.00 (1.15) ***	16.65–21.34	<0.001	19.59 (2.31) ***	14.67–24.52	<0.001

Notes: Source: National Survey of American Life (NSAL 2001–2003), Outcome: Discrimination (Everyday), Independent variables treated as interval measures. Household income measured as (1) 0–9999 USD, (2) 10,000 USD–19,999 USD, (3) 20,000 USD–39,999 USD, and (4) 40,000 USD or more. Education attainment measured as (1) equal or less than 11 years, (2) 12 years, (3) 13 to 15 years, and (4) 16+ years. Confidence Interval (CI); Standard Error (SE). ** *p* < 0.01, *** *p* < 0.001.

## Appendix B

**Table A2:** Summary of linear regression on the effects of educational attainment and household income on perceived discrimination in the pooled sample of African American and Non-Hispanic White men in the National Survey of American Life.

Characteristics	Total Sample (*n* = 1643)					
	Model 1			Model 2		
	b (SE)	95% CI	*p*	b (SE)	95% CI	*p*
Race (African Americans)	3.89 (0.56) ***	2.77–5.01	<0.001	0.07 (2.30)	−4.54–4.69	0.975
Age	−0.14 (0.01) ***	−0.16–0.12	<0.001	−0.14 (0.01) ***	−0.17–0.12	<0.001
Educational attainment	0.05 (0.37)	−0.70–0.79	0.901	−0.12 (0.58)	−1.29–1.05	0.835
Household income	−0.50 (0.21) *	−0.92–0.08	0.020	−0.90 (0.30) **	−1.51–0.30	0.004
Race × Educational Attainment	-	-		0.42 (0.68)	−0.94–1.78	0.537
Race × Household Income	-	-		0.80 (0.40) **	0.00–1.62	0.005
Intercept	17.17 (1.37) ***	14.42–19.92	<0.001	19.24 (2.14) ***	14.93–23.55	<0.001

Notes: Source: National Survey of American Life (NSAL 2001–2003), Outcome: Discrimination (Everyday), Independent variables treated as interval measures. Household income measured as (1) 0–9999 USD, (2)10,000 USD–19,999 USD, (3) 20,000 USD–39,999 USD, and (4) 40,000 USD or more. Education attainment measured as (1) equal or less than 11 years, (2) 12 years, (3) 13 to 15 years, and (4) 16+ years. CI: Confidence Interval; SE: Standard Error. * *p* < 0.05, ** *p* < 0.01, *** *p* < 0.001.
